# Bibliometric analysis of the reporting of p values in the summaries of anaesthetic publications

**DOI:** 10.1111/anae.70245

**Published:** 2026-05-26

**Authors:** Markus Huber, Alexander Fuchs

**Affiliations:** ^1^ Inselspital, Bern University Hospital University of Bern Bern Switzerland

Scientific manuscripts are the main channel through which research findings reach the wider community. The summary plays a central role in communicating the clinical importance of a study to its readers [1]. Frequentist statistical methods dominate biomedical research and reporting the significance of findings using p values has been a cornerstone of summaries in the biomedical and life science for decades [2]. The problems associated with the practice of significance hunting [3] and publication bias have long been recognised and debated [4].

Although the reporting of p values and over‐representation of statistically significant findings have been examined in fields such as reproductive medicine [3], a comparable analysis in anaesthesia is lacking. We therefore assessed the patterns of p value reporting in summaries within the anaesthesia literature using a bibliometric analysis. We placed particular emphasis on the order in which statistically significant and non‐significant results were presented, and on potential differences between observational studies and randomised controlled trials.

We searched PubMed using the medical subject headings major topics ‘anesthesia and analgesia’ and ‘anesthesiology’, filtered separately for the publication types ‘observational study’ and ‘randomized controlled trial’ for all publications up to and including 2025. For each publication, we extracted the publication year; article type; journal name; and summary. Each summary was screened for p values using regular expression matching, which allowed extraction of exact p values (e.g. p = 0.004) and threshold‐expressed p values (e.g. p < 0.001). Data were analysed using R (version 4.2.3, R Studio for Statistical Computing, Vienna, Austria) and PubMed queries were executed using the RISmed package [5].

The search identified 9538 publications and a summary could be extracted from 9427 (99%). Most studies (7961, 84%) were randomised controlled trials (Table 1). We found p values in 5515 (58%) of these. In total, 6578 (13%) and 45,271 (87%) p values were extracted in the summaries of observational studies and randomised controlled trials, respectively. Overall, 35,771 (69%) of the p values were statistically significant, more so in summaries of observational studies (5120, 78%) than for randomised controlled trials (30,651, 68%).

**Table 1:** Characteristics of summaries in the anaesthesia literature identified in the PubMed database and associated p value reporting. Values are number (proportion) or median (IQR [range]).

	All n = 9538	Observational study n = 1577 (17%)	Randomised controlled trial n = 7961 (84%)	p value
Medical subheading major topic				< 0.001
Anesthesia and analgesia	9321 (98%)	1484 (94%)	7837 (98%)	
Anesthesiology	217 (2%)	93 (6%)	124 (2%)	
Year	2018 (2014–2022 [2001–2025])	2020 (2017–2023 [2012–2025])	2018 (2013–2022 [2001–2025])	< 0.001
Summary present	9427 (99%)	1534 (97%)	7893 (99%)	< 0.001
p value(s) in the summary	5515 (58%)	708 (45%)	4807 (60%)	< 0.001
Number of p values	8 (4–12 [1–60])	8 (4–12 [1–48])	8 (4–12 [1–60])	0.359
Number of p values extracted	51,849 (100%)	6578 (13%)	45,271 (87%)	–
Type of p value presentation				0.166
Numerical (p = or p:)	28,830 (56%)	3605 (55%)	25,225 (56%)	
Smaller than a threshold (p <)	23,019 (44%)	2973 (45%)	20,046 (44%)	
Statistical significance				< 0.001
Non‐significant (p ≥ 0.05)	16,078 (31%)	1458 (22%)	14,620 (32%)	
Significant (p < 0.05)	35,771 (69%)	5120 (78%)	30,651 (68%)	

**Figure 1:**
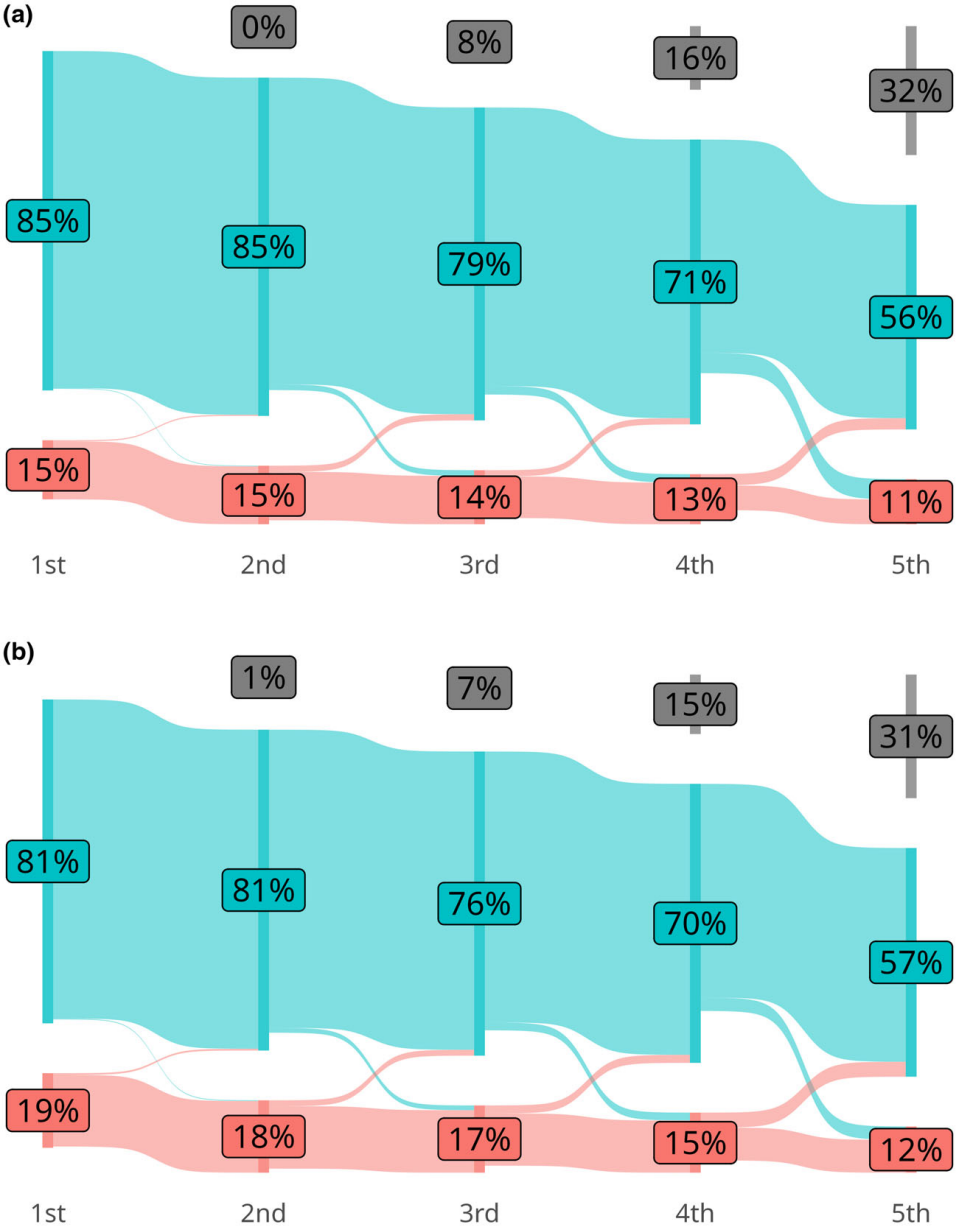
Sankey diagram illustrating the reporting of p values in summaries from the anaesthesia literature for: (a) observational studies; and (b) randomised controlled trials according to statistical significance (blue, p < 0.05; red, p ≥ 0.05; grey, no p value reported). Proportions indicate the proportion of statistically significant, non‐significant and unreported outcomes relative to their order of appearance in the summary. For example, 1.3% of summary of randomised controlled trials reported only a single p value and 6.8% reported only two p values. The number of reported outcomes was chosen for illustration purposes only; the first five reported outcomes seemed reasonable to study and illustrate the sequential pattern of p value reporting.

We found a clear preference for reporting statistically significant values: 603 (85%) of the first p values reported in the summaries of observational studies and 3907 (81%) in summaries of randomised controlled trials were statistically significant (Fig. 1). Most summaries continued to report significant p values, and a non‐significant p value rarely followed a significant one, or vice versa. On average, the p values < 0.05 were 5.1 times more common among the first five reported outcomes than p values > 0.05. This ratio was 5.6 in observational studies and 4.6 in randomised controlled trials.

Our bibliometric analysis of summaries published over the past 25 years shows that research in anaesthesia strongly favours the reporting of statistically significant findings. Overall, 35,771 (69%) of the p values reported in the summary were statistically significant. A novel aspect of our work is the explicit consideration of the order in which p values appear in summaries. This ordering reveals a cascade of statistically significant results, as once a significant result is reported it tends to be followed by further statistically significant ones (Fig. 1), with p values < 0.05 appearing approximately five times more often than p values ≥ 0.05. Our results suggest that the general notion applies that it is often reasonably safe to assume that results presented in the summaries of anaesthetic research are statistically significant [6]. This pattern reflects the well‐recognised problems of publication bias, and the risk of false‐positive findings from an emphasis on p < 0.05 should always be considered [7].

The generalisability of our results is limited by our reliance on a single bibliometric database and specific search terms, as well as the accuracy of our p value extraction algorithm. For instance, primary and secondary outcomes could not be distinguished. We also focused exclusively on p values and did not address the broader discussion around effect size measures and their uncertainty, which some journals now advocate in place of, or alongside, p values [8]. The derivation of p values from summaries reporting 95%CIs only was beyond the scope of this study. As medical subject headings terms are assigned in American English by the National Library of Medicine, UK spelling variants were not searched separately, though medical subject headings indexing is standardised regardless of journal spelling conventions.

Summaries in the anaesthesia literature show a strong tendency to report statistically significant findings, both in observational studies and randomised controlled trials. Summaries that report a p value < 0.05 are approximately five times more likely to continue reporting p values below this conventional threshold.
